# Surgical stress: the muscle and cognitive demands of robotic and laparoscopic surgery

**DOI:** 10.1097/AS9.0000000000000284

**Published:** 2023-04-28

**Authors:** Abdul Shugaba, Daren A. Subar, Kate Slade, Mark Willett, Mohammed Abdel-Aty, Iain Campbell, Nick Heywood, Louis Vitone, Adnan Sheikh, Mike Gill, Bachar Zelhof, Helen E. Nuttall, Theodoros M. Bampouras, Christopher J. Gaffney

**Affiliations:** 1 Lancaster Medical School, Lancaster University, UK; 2 East Lancashire NHS Hospitals Trust, UK; 3 BRIDGES Research Group, Department of General Surgery, Royal Blackburn Teaching Hospitals NHS Trust; 4 Department of Psychology, Lancaster University, UK; 5 Lancashire Teaching Hospitals NHS Foundation Trust, UK; 6 School of Sport and Exercise Sciences, Liverpool John Moores University, UK

**Keywords:** Laparoscopic surgery, Robotic surgery, Minimally invasive surgery, Electromyography, Electroencephalography

## Abstract

**Introduction:**

Surgeons are among the most at-risk professionals for work-related musculoskeletal decline and experience high mental demands. This study examined the electromyographic (EMG) and electroencephalographic (EEG) activities of surgeons during surgery.

**Methods:**

Surgeons who performed live laparoscopic (LS) and robotic (RS) surgeries underwent EMG and EEG measurements. Wireless EMG was used to measure muscle activation in four muscle groups bilaterally (biceps brachii, deltoid, upper trapezius, and latissimus dorsi), and an 8-channel wireless EEG device was used to measure cognitive demand. EMG and EEG recordings were completed simultaneously during (i) noncritical bowel dissection, (ii) critical vessel dissection, and (iii) dissection after vessel control. Robust ANOVA was used to compare the %MVC_RMS_ and alpha power between LS and RS.

**Results:**

Thirteen male surgeons performed 26 laparoscopic surgeries (LS) and 28 robotic surgeries (RS). Muscle activation was significantly higher in the right deltoid (p = 0.006), upper trapezius (left, p = 0.041; right, p = 0.032), and latissimus dorsi (left, p = 0.003; right, p = 0.014) muscles in the LS group. There was greater muscle activation in the right biceps than in the left biceps in both surgical modalities (both p = 0.0001). There was a significant effect of the time of surgery on the EEG activity (p <0.0001). A significantly greater cognitive demand was observed in the RS than in the LS with alpha, beta, theta, delta, and gamma (p = 0.002 – p <0.0001).

**Conclusion:**

These data suggest greater muscle demands in laparoscopic surgery, but greater cognitive demands in robotic surgery.

## Introduction

Surgical procedures have evolved in recent years to improve short- and long-term patient outcomes^(1)^. Minimally invasive surgery is an integral part of this procedure. This principally involves either the standard laparoscopic or robotic technique. Minimally invasive surgical incisions are smaller than those in conventional open surgical techniques. Furthermore, there is less tissue manipulation, reduced blood loss, and reduced catabolic response^(2)^, ultimately leading to improved patient outcomes^(3)^. Standard laparoscopic surgery (LS) instruments and techniques are the mainstay of minimally invasive surgery. However, over the last 20 years, robotic surgery (RS) has gained popularity in several surgical specialties, with a year-on-year increase in uptake^(4)^.

The basic difference between RS and LS lies in the design of the instruments and stability of the robotic platform used in performing surgical procedures. Most laparoscopic instruments are not wristed and offer only four degrees of motion across a port that serves as a fulcrum^(5)^. In addition, two-dimensional monitor systems are still predominantly used in LS to display images of the operating field and therefore limit depth perception^(6)^. The limitations associated with the standard LS instrument design, along with the requirement for surgeons to maintain non-ergonomic postures for prolonged periods while operating, place them at risk of increased musculoskeletal strain. The most affected muscle groups include the back, neck, lower extremities, and shoulders, with a prevalence of 73–90% ^(7, 8)^. In addition, the cumulative effect of repetitive strain injuries is reported to be 73–100% for standard laparoscopy^(9)^.

RS affords better ergonomics during the operation in comparison to LS. Owing to their endo-wristed instruments, RS provides three additional degrees of motion^(10)^. These systems also filter out physiological tremors thereby making movements more precise^(11, 12)^. Additionally, the robotic system allows surgeons to perform procedures while sitting comfortably with an arm rest, providing a natural working axis, thereby improving ergonomics ^(13)^. Additionally, the da Vinci™ robotic system offers an immersive experience through its three-dimensional viewing cart, which improves stereoscopic depth perception ^(6)^.

In addition to muscle demands, surgeons experience cognitive demands during surgery. This cognitive workload increases with complex and prolonged operating procedures^(14)^. One component of this increase in cognitive demand is related to musculoskeletal stress. With increased musculoskeletal demand, the central nervous system responds by activating a greater number of motor neurons or increasing their discharge rate^(3)^, resulting in feelings of higher effort exertion to maintain muscle contraction^(15)^. Simulation-based studies using validated questionnaires and electromyography (EMG), reported that RS is associated with decreased musculoskeletal demands compared to LS^(13, 16)^. Therefore, it is possible that RS requires less cognitive demands than LS. However, questionnaire-based data have also shown RS to require a higher level of alertness and awareness^(17)^ because currently, the majority of RS operations being performed are for more complex or challenging cases. There also appears to be elevated eye strain in RS compared to other surgical modalities^(18)^. Studies that have examined muscle and cognitive demands in the LS and RS^(19)^ have mostly been conducted in simulated settings, and none have measured them simultaneously.

This study aimed to objectively determine the muscle and cognitive demands of surgeons performing live laparoscopic and robotic surgery.

## Materials and Methods

### Study ethics and participants

This study was approved by the Faculty of Health and Medicine Research Ethics Committee of Lancaster University (FHMREC- 19052) and the National Health Scheme Research Ethics Committee (NHSREC- 20/ES/0081) and was registered at Clinicaltrials.gov (NCT04477746). All study participants provided written informed consent before experimentation and all experiments were conducted in accordance with the *Declaration of Helsinki*.

### Design

Consultant surgeons across three specialties (colorectal surgery, urology, and gynaecology) at two National Health Service (NHS) foundation trusts in the UK (East Lancashire NHS Hospital Trust and Lancashire Teaching Hospital NHS Foundation Trust) were invited to participate. All surgeons had certificates of completion of training (CCT) and routinely performed laparoscopic and/or robotic procedures.

Following consent, surgeons trained in LS were assigned to the *laparoscopic* (LS) group and those trained in RS were assigned to the *robotic* (RS) group. Surgeons with significant musculoskeletal or mental health conditions or symptoms were excluded from this study. To ensure uniformity and comparability, specific types of surgeries using either LS or RS were performed within each specialty. These provided similar points of interest (POI) (Table 1) when surgeons performed identical tasks required during the procedures, thereby providing a uniform basis for comparison. Surgical procedures, including colorectal surgery (anterior resection, Hartmann’s, and right colectomies), urology (partial and radical nephrectomies), and gynaecology (hysterectomies), were identified in the surgeon’s operating diaries. On the day of the procedure, the patients (operated upon) provided written informed consent.

### Procedures

All surgeries, whether LS or RS, were conducted using standard operating procedures, with the addition of wireless EMG and electroencephalography (EEG) sensors to the surgeon. The placement of both wireless devices did not interfere with the surgeons’ ability to operate.

#### Laparoscopic surgery

Surgeons performed surgery using the laparoscopic stack system available in their operating theatres with their preferred instruments (graspers, scissors, staples, energy-delivering dissectors, and a zero- or thirty-degree angled camera). Surgeons placed laparoscopic ports to introduce a camera and laparoscopic instruments and had a trained assistant or trainee navigating the laparoscopic camera, with clear instructions from the surgeon when required. The stack system and monitor were placed at the surgeons’ perceived optimal ergonomic height and distance from the operating table, respectively. The surgeons remained standing while operating, ensuring that the operating table was at an optimal height based on each surgeon’s preference.

#### Robotic surgery

The surgeons used the robotic systems available in their theatres (da Vinci^®^ Xi; Intuitive, Sunnyvale, California, USA and da Vinci^®^ X; Intuitive, Sunnyvale, California, USA). Depending on the surgical procedure, surgeons use three or four robotic arms on the patient’s cart. A trained assistant or surgical trainee assisted with instrument transfers on the patient cart with clear instructions from the surgeon. The instruments utilised were Standard graspers, scissors, and staples were used. Surgeons performed the procedures at the console with their chair and arm rests adjusted to perceived optimal ergonomic heights.

### Electromyography (EMG)

Muscle activity was recorded bilaterally using EMG from four muscles: biceps brachii (arms), deltoid (shoulder), upper trapezius (neck), and latissimus dorsi (back). These muscles are the muscles most at risk for fatigue ^(20, 21)^. The skin overlying the placement area was prepared by shaving and cleansing with a 70% isopropyl alcohol pad. EMG surface wireless sensors with a 10 mm inter-electrode distance (Trigno, Delsys, Inc., Boston, MA, US) were then placed on these sites according to SENIAM (Surface EMG for a Non-Invasive Assessment of Muscles)^(22)^ and other recommendations^(23)^. The surgeons performed one isometric maximal voluntary contraction (MVC) of each muscle group prior to commencing the surgery, and EMG was recorded. During the predefined points of interest (POI), 120 s of EMG recordings were performed (Table 1). In the RS group, these POI were present in portions of the surgical procedures performed with the assistance of the robotic console. The raw EMG signal was collected at a sampling rate of 2000 Hz and filtered using high- and low-pass filters of 10 and 500 Hz, respectively. Next, the signal was smoothed using the root mean square (RMS) over 150ms envelopes (EMG Works, Delsys Inc., Boston, MA, USA). Finally, the average RMS EMG from the points of interest was normalized to isometric MVC EMG and computed as %MVC_RMS_.

### Electroencephalography (EEG)

**Figure 1:**
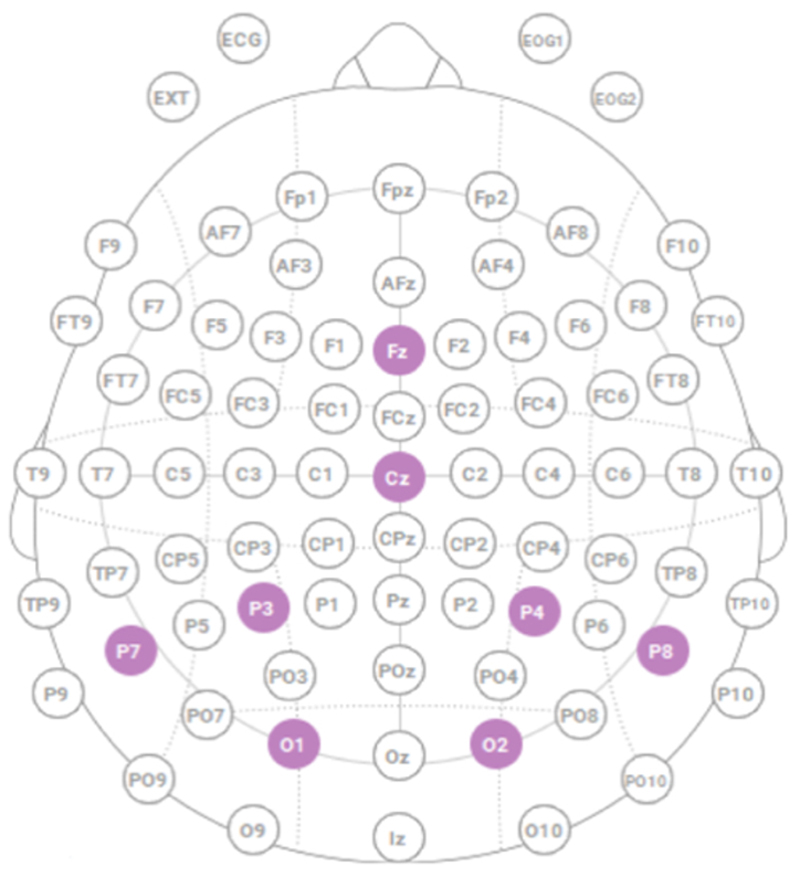
Electroencephalogram (EEG) electrode placement

An Enobio 8 5G wireless device (Neuroelectrics, Cambridge, MA, USA) was used to record EEG data from eight channels (Cz, Fz, P7, P8, P3, P4, O1, and O2) according to the international 10–20 Montage system^(24, 25)^, as depicted in Figure 1, with a reference electrode connected to the right ear lobe^(24, 25)^. These were silver–silver chloride (Ag|AgCl) electrodes, which were embedded in a head cap that the surgeons wore prior to scrubbing for surgery. A semi-solid electrode gel was applied between the scalp and the electrodes to facilitate signal detection. Data were initially visualized at a sampling rate of 500 Hz, and power line noise (50 Hz) was removed by enabling the band-reject filter and removal of electrooculogram (EOG) artifacts by choosing an EMG filter in ENOBIO NIC1.4 software (Neuroelectrics, Barcelona, Spain). The EEG device was then placed in Holter mode and similar to the EMG data, the time corresponding to the various POI, including the baseline, was matched (Table 1).

The acquired EEG signals were subjected to pre-processing in EEGLAB, a MATLAB toolbox (Mathworks, Natick, MA, USA)^(26)^. The signal was downsampled to 256 Hz, re-referenced to the average of all channels, and then high (0.1 Hz) and low-pass (40 Hz) filtering was applied. Data were segmented into four time points of interest. At each time point, the power in each frequency band of interest was calculated using the EEGLAB spectopo() function^(27)^. MATLAB’s pwelch() function was used to calculate the power spectral density (PSD) using the default settings: a Hamming window of 400 ms with 50% overlap. The average power for each frequency band was then calculated for each of the eight channels by obtaining the average power frequency across individual channels with a unit of power of 10 × log(microvolt^2)/Hz. The power was then averaged across all channels to obtain the power spectral density across the full-electrode montage.

The signal-to-noise ratio (SNR) was calculated to gauge the EEG signal quality across recordings. We obtained two versions of the EEG signals for each time point of interest. In one version, the EEG signal was subjected to all aforementioned pre-processing steps, which we will refer to as the filtered signal, and in the other version, the EEG signal was subjected to all aforementioned pre-processing steps except low-pass filtering at 40 Hz, which we will refer to as the unfiltered signal. We calculated the root mean square (RMS) of the filtered and unfiltered EEG signals and the subsequent SNR for each time point of interest using the following equation:

$$SNR=20log_{10}(\frac{\sqrt{\;mean(unfilteredEEGsignal{\;}^{2}\text{)}}}{\sqrt{\;mean(filteredEEGsignal^{2}\text{)}}})$$

### Statistical analysis

The normality of EMG %MVC_RMS_ and EEG peak alpha amplitude data was determined using the Shapiro-Wilk test, and data were found to be normally distributed. A robust analysis of variance (ANOVA)^(28, 29)^ with subsequent Tukey corrections was applied to both EMG %MVC_RMS_ and EEG peak alpha amplitude, which were then used to compare differences between LS and RS across various points of interest. Unpaired t-tests were performed on continuous demographic variables, the duration of surgery, glove size and years of experience to compare the LS and RS groups. All statistical analyses and figure creation were performed using GraphPad Prism v9.4.1 (GraphPad Software, San Diego, Calif., USA). Data are presented as the mean ± SEM, unless otherwise stated. Statistical significance was set at p < 0.05.

## Results

### Comparison of LS and RS procedures

Thirteen healthy male surgeons were enrolled in the study, of which seven were LS surgeons and six were RS surgeons (Table 2). The surgeons performed 26 LS and 28 RS procedures (total number of surgeries, n = 54) (Table 3). The mean operative times were similar between LS (mean ± SE) (112 ± 10 mins) and RS (116 ± 8 mins) (p >0.05). Six procedures were converted to open surgery (four LS, two RS) and were subsequently excluded from the analysis. Surgeons performing LS had significantly greater experience (p = 0.009). There was no difference in glove size between both groups (p = 0.582).

### Muscle activity

#### LS is associated with greater muscle activation than RS

**Figure 2:**
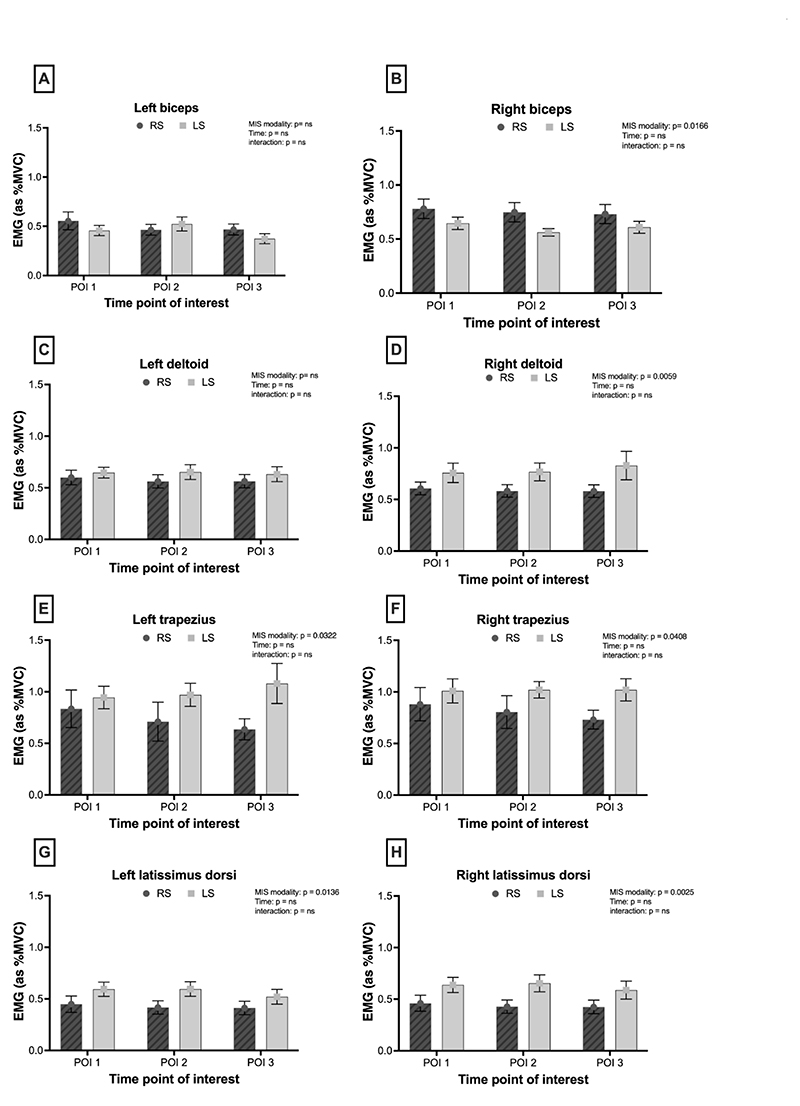
RMS EMG data of muscle activation of the left (left Panel) and right (right Panel) between robotic (filled bars) versus laparoscopic (empty bars) surgery. Data are presented as means + SEM. **(A)**. EMG activity across time in the right biceps **(B).** EMG activity across time in the left biceps **(C).** EMG activity across time in the right deltoid **(D).** EMG activity across time in the left deltoid **(E).** EMG activity across time in the right trapezius **(F).** EMG activity across time in the left trapezius **(G).** EMG activity across time in the right latissimus dorsi **(H).** EMG activity across time in the left latissimus dorsi.

Only the right biceps showed increased muscle activation in the RS group (Figure 2A and 2B; p = 0.017). The left deltoid did not show any significant difference; however, there was a greater muscle demand in the right deltoid within the LS group across all three points of interest, corresponding to initial dissections, dissection along major vessels, and organ manipulation after vessel control (Figure 2C and 2D; left, p >0.05; right, p = 0.006). The lower trapezius showed greater muscle activity in the LS group (left, p = 0.032; right, p = 0.048), more prominently during vessel dissection and manipulation of tissues of the target organ being operated on (POI 2 and 3) (Figure 2E and 2F). Muscle activity in the latissimus dorsi was increased in the LS group (left, p = 0.014; right, p = 0.003; Figure 2G and 2H).

#### Bilateral asymmetry in muscle activity

**Figure 3:**
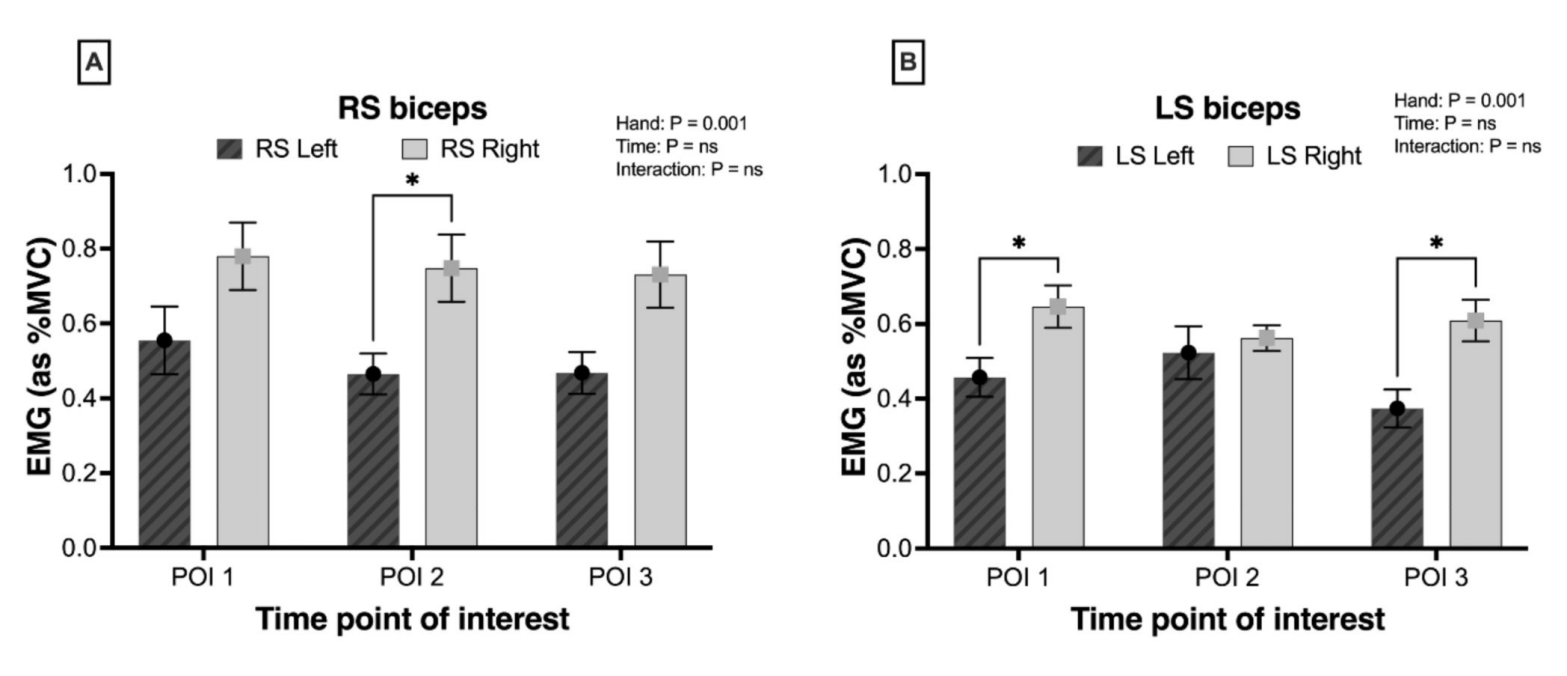
RMS EMG data of muscle activation of the left (filled bars) and right muscle (empty bars) groups in the robotic and laparoscopic surgery groups. **(A)**. EMG activity across time within RS group in the biceps muscle **(B)**. EMG activity across time within LS group in the biceps muscle. * p < 0.05

We investigated the differences in %MVC_RMS_ between the left and right muscles to determine any bilateral asymmetry in the muscle activation. Both LS and RS groups displayed significantly greater muscle activation in the right biceps brachii (Figure 3A and 3B, both p = 0.001). Similar levels of muscle activation were observed between the left and right sides of the deltoid, upper trapezius, and latissimus dorsi across all POI in both the LS and RS (p >0.05).

### EEG Analysis

#### Cognitive demand is greater with RS

**Figure 4:**
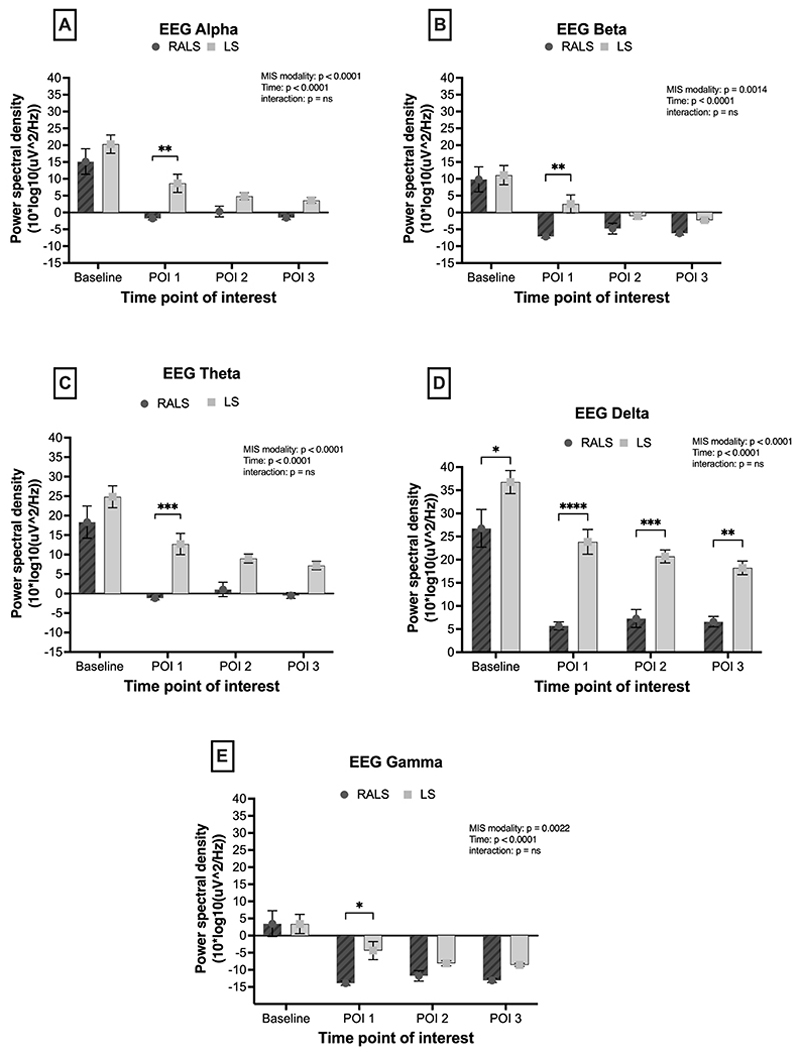
Alpha power indicated there was greater overall cognitive demand in RS (dark grey bars) relative to LS (light grey bars). In both RS and LS, cognitive demand increased in surgery relative to baseline, and was sustained throughout surgery. Exploratory analyses of EEG activity in different frequency bands reflected similar observations. **(A)**. Alpha power activity across time **(B).** Beta power activity across time **(C).** Theta power activity across time **(D).** Delta power activity across time **(E).** Gamma power activity across time. * p < 0.05; **p < 0.01; *** p < 0.001; **** p < 0.0001.

Power spectral density in the alpha frequency band (8–13 Hz) was used as a measure of cognitive demand during (POI 1) initial dissection, (POI 2) dissection along major vessels, (POI 3) organ manipulation after vessel control, and at baseline immediately before surgery, in both LS and RS (Figure 4A). Here, alpha power is used as a proxy for attention, whereby greater attentional demand is indicated by lower alpha (alpha desynchronization). A mixed- model ANOVA indicated a significant effect of modality on alpha power, whereby alpha power was significantly lower during RS relative to LS (p <0.001), indicating overall greater attentional demand in RS relative to LS. There was also a significant effect of time on alpha power; in both modalities, there was a significant reduction in alpha power from baseline to surgical time points: during initial dissection around the target organ, alpha power decreased from baseline and remained decreased during dissection along major vessels and post-vessel control manipulation of the target organ (p <0.001). The interaction between time and modality was not significant (p > 0.05).

An exploratory analysis compared EEG data from other frequency bands in both the RS and LS groups. The power spectral density in the beta band (Figure 4B) was lower in RS than in LS, which may indicate a fatigue effect, wherein RS leads to higher cognitive fatigue (p = 0.001). Theta power (Figure 4C) was lower in the RS than in the LS (p < 0.001). Delta power (Figure 4D) was lower in RS than in LS (p < 0.001) and gamma power (Figure 4E) was lower in RS than in LS (p = 0.002). All frequency bands indicated a significant effect of time, whereby EEG power was reduced during surgery relative to baseline (p < 0.001).

To evaluate the EEG signal quality across the recording during surgery, the signal-to-noise ratio (SNR) between the unfiltered and filtered EEG signals was calculated at each time point. One-way ANOVA indicated that there was no significant difference in SNR across the four time points (*p* > 0.05).

An exploratory analysis was conducted to test whether there was a relationship between the observed musculoskeletal demands and the cognitive demands experienced by surgeons. For this, EEG changes over time, computed as the difference between alpha power at baseline and the last surgical time point (POI 3; Table 1) for both LS and RS, were correlated with the corresponding EMG %MVC RMS data for the trapezius and latissimus dorsi, which displayed the greatest muscle demand. A significantly weak correlation between EEG alpha wave desynchronization and right trapezius muscle demand in the LS group was noted (p = 0.023, r^2^ = 0.39).

## Discussion

This study demonstrated an increased muscle demand in the shoulder, neck, and lower back when surgical procedures were performed using the standard laparoscopic approach versus the robotic platform. The study also demonstrated increased cognitive demand in the RS, as evidenced by the EEG. To the best of our knowledge, this is the first study to simultaneously compare the muscle and cognitive demands of real-life surgeries using objective measures.

### Muscle demand is lower in robotic than standard laparoscopic surgery

Several studies have demonstrated that laparoscopic surgery increases musculoskeletal demand compared to open surgery^(30–32)^; especially affecting the muscles of the shoulder, neck, and back. Recent studies have suggested that RS reduces musculoskeletal demand compared with LS^(33)^. The present findings are similar to those of comparative simulation studies that used questionnaires^(34, 35)^ and EMG data^(19)^ to measure musculoskeletal demands. Furthermore, these findings are echoed in a recent systematic review showing increased demand associated with LS^(33)^. These findings can be explained by the design of the robotic console, which has an arm rest^(36)^. This arm rest supports the weight of the surgeons’ upper limbs, and thereby distributes the force exerted on their shoulders. The shoulder muscles provide a fulcrum for the arm^(37)^, and because laparoscopic surgeries are predominantly performed while standing, shoulder muscle fatigue more easily attempts to fix and stabilize the arm to improve accuracy. In contrast, the fixed point in RS is transferred to multiple sites (forearm, seat, and headrest); therefore, shoulder stabilization becomes less essential for task execution.

Surgeons experience increased muscle demands on the lower back because of the requirement to stand and perform standard laparoscopic surgery, often with the stack system monitor placed at nonergonomic angles^(38)^. However, with RS, surgeons comfortably sit on a chair at the console, supporting their body and pelvis, thereby limiting stress on the back muscles. The robotic console has a headrest on the viewing cart for the surgeons to rest their forehead. As a result, the requirement of the neck muscles to support the head while viewing a 3-dimensional image of the operating field is reduced^(39)^, which is reflected in our neck muscle results.

The difference in muscle demand was sustained throughout the surgery; therefore, there was constantly greater muscle demand in laparoscopic surgery. Indeed, this increase in muscle demand associated with laparoscopic surgery is associated with a high rate of musculoskeletal injuries and pain among surgeons^(8)^. Studies have reported a high prevalence of musculoskeletal complaints, with up to 37.5% of surveyed surgeons being on either medication or another form of therapy ^(40)^. Decreased levels of muscle activation associated with RS may help reduce the risk of work-related musculoskeletal injuries.

### Robotic surgery is associated with increased cognitive demand

The nature of the surgery has led to sustained attention. In addition, elevated muscle demand requires greater cortical motor output to sustain the peripheral muscle activity. The EEG activity in different frequency bands is related to different brain states. High alpha power is typically observed when the brain is ‘idle’ in a phase of restful wakefulness, and this then decreases when brain activation increases ^(41)^. The decrease in alpha power is due to amplitude changes within the alpha band when oscillations become out-of-phase, that is, desynchronized, when a task or event is being performed. This process is termed event-related desynchronization (ERD) ^(42, 43)^. Based on the alpha wave activity, this study found that surgeons maintained sustained, focused attention throughout surgery relative to baseline in both specialties. Indeed, there is a constant increase in cognitive load in the RS, which does not result in higher muscle activation, as originally hypothesized.

Alpha event related desynchronisation was significantly greater in RS. Studies have shown that alpha activity correlates with structural and functional connectivity within the primary visual cortex^(44)^ and is related to the activity of thalamocortical neurons, which facilitates the transfer of visual information to the cortex^(45)^. This suggests that RS may place a greater demand on visual attention systems. In RS, the surgery is viewed in 3-dimensions through a binocular lens, which offers the additional advantage of blocking distracting visual stimuli in the theatre environment. Another explanation is that heightened visual attention is required to safely manipulate tissues to compensate for the loss of haptic feedback from the robot. This concept has been described as “visual haptics” in robotic surgery^(46)^.

With the alpha desynchronization observed in both groups reflecting a state of sustained focused attention, there was also an accompanying similar desynchronization in the beta frequency band. The reduction in beta power was greater in the RS group. This suggests that the attentional demands required to perform RS may lead to fatigue. Beta desynchronization has been observed during mental fatigue in drivers^(47, 48)^, sustained visual search^(49)^, and mental arithmetic^(50)^. Unlike standard cognitive assessments or attention tasks, engagement in surgical procedures is perhaps a unique situation that demands high levels of attention toward varied and stimulating mental tasks for sustained periods, inducing feelings of fatigue. This could explain the simultaneous observation of alpha and beta desynchronization, indicating high cognitive demand and increased mental fatigue in surgeons when performing surgery.

### Strengths and Limitations

These data were recorded in a naturalistic setting and impedances could not be altered throughout the surgical procedure. Signal-to-noise ratios were consistent across all points of interest, suggesting that any decrease in power across the alpha and beta frequency bands did not reflect methodological confounding. Second, the procedures completed using laparoscopy and robotic surgery were performed differently. Therefore, it is impossible to have identical comparisons across specialties in real-life surgical environments.

Due to the limited surgeon numbers at the recruitment sites, a repeated measures design using a within-subject approach was not possible for the present study. However, future studies should consider this design as it will limit the heterogeneity associated with a between-subject design.

Only male surgeons were able to participate in the study. This unintended single-sex recruitment was likely a fair representation of the male-dominated profession^(51)^. As females could show different characteristics which could affect muscular and cognitive demands^(52)^, the present results cannot be extrapolated to both sexes. Future studies should explore sexbased differences in muscular and cognitive demands amongst surgeons.

### Summary and Conclusion

The purpose of this study was to determine the muscle and cognitive demands of laparoscopic and robotic surgery. In conclusion, robotic surgery was associated with a reduction in musculoskeletal demands, particularly on the muscles of the surgeon’s right-hand side, as demonstrated by lower EMG activity in the deltoid, lower trapezius, and latissimus dorsi muscles. Collectively, these findings indicate a lower risk of musculoskeletal injury after robotic surgery. However, an associated increased cognitive demand was observed in both surgical modalities with time (reduced alpha power), which was significantly greater in robotic surgery. Further research is required to investigate how these observations are affected by the duration and number of surgeries performed and whether such sustained focused attention leads to long-term cognitive fatigue.

## Figures and Tables

**Table 1:** Points of Interest across specialties

Colorectal	Urology	Gynaecology
1. Zeroing	1. Zeroing	1. Zeroing
2. Bowel Mobilisation	2. Bowel Mobilisation	2. Round Ligament dissection
3. Vessel dissection & control	3. Vessel dissection & control	3. Vessel dissection & control
4. Mesenteric dissection	4. Kidney Mobilisation	4. Colpectomy

**Table 2:** Surgeon Demographics for both groups. Data presented as mean ± SEM

		RS(n = 7 surgeons)	LS(n = 6 surgeons)	*p*values
**Age (years)**		46 ± 1.52	49 ± 3.42	0.418
**Body mass (Kg)**		91 ± 1.83	92 ±3.14	0.785
**Height (m)**		1.72 ± 0.04	1.75 ± 0.03	0.620
**BMI (kg/m^2^)**		31 ± 1.48	30 ± 0.51	0.622
**Handedness (Left: Right)**		2:5	1:5	n/a
**Average Glove size**		7.5 ± 0.11	7.5 ± 0.13	0.582
**Muscle pain in the past year**	**Neck**	None	1	n/a
	**Shoulder**	1	None	n/a
	**Lower Back**	4	3	n/a
**General health**		All reported “very good health”	All reported “very good health”	n/a
**Years of Experience**		4 ± 0.4	8 ± 1.04	0.009

**Table 3:** Distribution of procedures

	RS				LS			
Procedure type	Partial Nephrectomy	Radical Nephrectomy	Right Colectomy	Anterior Resection (Without TME)	Right Colectomy	Hartmann’ s	Anterior Resection (Without TME)	Hysterectomy
**Procedures (n)**	14	5	1	8	6	2	6	12
**Mean operating time** ± **SEM (mins)**	108 ± 11	126 ± 19	49 (n/a)	95 ± 14	138 ± 14	114 ± 25	187 ± 47	75 ± 6
**Time (am:pm)**	5:2	3:2	1:0	8:0	4:2	2:0	6:0	6:6

## Data Availability

The data supporting the findings of this study are available from the corresponding author (CG) upon reasonable request.
